# Fasting Glucose for the Diagnosis of Gestational Diabetes Mellitus (GDM) during the COVID-19 Pandemic

**DOI:** 10.3390/nu14163432

**Published:** 2022-08-20

**Authors:** Nieves Luisa González González, Enrique González Dávila, Fernando Bugatto, Begoña Vega-Guedes, Pilar Pintado, L. Tascón, Nazaret Villalba Martin, Walter Plasencia, Ana Megía

**Affiliations:** 1 Obstetrics and Gynaecology Department, University of La Laguna, 38200 San Cristobal de La Laguna, Spain; 2 Spanish Diabetes and Pregnancy Study Group, Spanish Section of Perinatal Medicine (SEMEPE) of Spanish Society of Spanish Society of Gynecology and Obstetrics (SEGO) and Spanish Society of Diabetes (SED), 28002 Madrid, Spain; 3 Mathematics, Statistics and Operations Research Department, IMAULL. University of La Laguna, 38200 La Laguna, Spain; 4 Division of Maternal-Fetal Medicine, Obstetrics and Gynecology Department, Puerta del Mar University Hospital, University of Cádiz and Biomedical Research and Innovation Institute of Cádiz (INiBICA), 11009 Cádiz, Spain; 5 Obstetrics and Gynaecology Department. Mother and Child Unisersity Hospital of the Canary Islands, 35016 Las Palmas de Gran Canaria, Spain; 6 Obstetrics and Gynaecology Department, Gregorio Marañón University General Hospital, 28007 Madrid, Spain; 7 Obstetrics and Gynecology Department, Canary Islands University Hospital, 38320 La Laguna, Spain; 8 Joan XXIII University Hospital, Tarragona, IISPV, Universitat Rovira i Virgili, 43005 Tarragona, Spain

**Keywords:** COVID-19 pandemic, gestational diabetes, gestational hyperglycemia, fasting glucose, diagnosis, early gestational diabetes, first trimester, perinatal outcomes

## Abstract

Background: During the COVID-19 pandemic, different non-validated tests were proposed to simplify the diagnosis of gestational diabetes (GDM). Aim: To analyse the effects of replacing the two-step approach for Early-GDM and GDM diagnosis, with a fasting plasma glucose test. Material and Methods: This is a cohort study consisting of 3200 pregnant women: 400 with Early-GDM, 800 with GDM and 2000 with Non-GDM diagnosed using the two-step approach. Using fasting plasma glucose for Early-GDM and GDM diagnosis, according to the recommendations of Spain, Australia, Italy and the UK during the pandemic, the rates of missed and new Early-GDM and GDM were calculated and perinatal outcomes were analysed. Results: Using fasting plasma glucose in the first trimester >100 mg/dL for Early-GDM diagnosis, the rates of post-COVID missed and new Early-GDM were 79.5% and 3.2%, respectively. Using fasting plasma glucose at 24–28 weeks <84 or >92, 95 or 100 mg/dL for GDM diagnosis, the rates of missed GDM were 50.4%, 78%, 82.6% and 92.4%, respectively, and 8.6%, 5.6% and 2.3% women with Non-GDM were diagnosed with new GDM. Conclusion: Fasting plasma glucose is not a good test for the diagnosis of GDM either in the first trimester or at 24–28 weeks.

## 1. Introduction

Given the unprecedented health emergency created by the COVID-19 pandemic, different authors and Scientific Societies [1,2,3,4,5,6,7] have proposed urgent recommendations to simplify the diagnosis of GDM and to avoid the contacts and prolonged presence in the labs required by the oral glucose tolerance tests (OGTTs), thus reducing the risk of infection among pregnant women. Analytical options include HbA1c, random plasmatic glucose (RPG) and/or fasting plasma glucose (FPG). The different recommendations and diagnostic thresholds proposed are shown in Table 1.

The WHO [8], the American College of Obstetricians and Gynaecologists [9] and the American Association of Diabetes (ADA) [10] have not made any specific recommendations for the diagnosis of GDM during the pandemic. 

It has been explicitly recognised that the recommendations proposals are urgent measures taken during an unprecedented health situation and lacking supportive scientific evidence, that ought to be implemented temporarily while OGTTs cannot be safely performed. However, the optional tests are less time-consuming, easy to perform, cheaper, and more convenient for pregnant women, and it could be considered to keep them as permanent diagnostic criteria for GDM. 

Previous studies have explored how the criteria recommended during the COVID-19 pandemic work compared with the one-step approach according to the International Association of Diabetes and Pregnancy Study Groups (IADPSG) criteria or the National Institute of Health and Clinical Excellence (NICE) recommendations [11,12,13,14,15]. 

To the best of our knowledge, the effectiveness of the optional tests has not been compared with that of the two-step strategy, which is the approach used in the clinical trials showing a benefit of treatment for GDM [16,17]. 

The objective of this study is to analyse the effects of replacing the tow-step approach for Early-GDM and GDM diagnosis, with a fasting plasma glucose test in the first trimester (1t-FPG), and at 24–28 weeks of pregnancy (2t-FPG), respectively, according to the different FPG thresholds recommended during the COVID-19 pandemic. 

## 2. Material and Methods

This multicentre retrospective cohort study was an initiative of the Spanish Diabetes and Pregnancy Study Group performed at five tertiary university hospitals in Spain. Medical records were reviewed to identify the first 400 pregnant women (80 in each hospital) with GDM diagnosed before 12 weeks of pregnancy (Early-GDM), who gave birth between 1 January 2018 and 31 December 2019 in each of the five hospitals (Early-GDM group). The sample size was set for the Early-GDM group with the intention of finding a precision of at least 7% in the proportion of adverse perinatal outcomes with the non-GDM group, with a confidence level of 95%, a power of 80%, an estimated proportion of 30% in the non-GDM group and 3% loss or lack of information. The GDM and non-GDM groups were constituted according to a 1:2:5 design. For each eligible woman, the next two consecutive women with GDM diagnosed at the 24–28 weeks of pregnancy (GDM group), and the next five pregnant women with normal glucose tolerance (Non-GDM group) were included. Thus, the study was carried out in a total of 3200 pregnant women, 400 with GDM diagnosed in the first trimester (Early-GDM group), 800 with GDM (GDM group) and 2000 women who showed normal glucose tolerance during pregnancy (Non-GDM group). 

Early-GDM, GDM and Non-GDM were defined according to the National Diabetes Data Group (NDDG) criteria [18], using the two-step approach and 100 g-OGTT with two or more values above 105, 190, 165 and 145 mg/dL after 0, 60, 120 and 180 min, for the Early-GDM or GDM diagnosis, according to the time of diagnosis, first trimester or 24–28 weeks of pregnancy. All pregnant women with Early-GDM and GDM received treatment with diet and exercise and/or insulin according to the recommendations of the Spanish Group of Diabetes and Pregnancy [19]. 

Inclusion criteria were knowing 1t-FPG, 2t-FPG and O’Sullivan test and OGTT values recorded when indicated, singleton pregnancy, delivery after 24 weeks of gestation, birth weight > 500 g. All cases with pre-gestational or overt diabetes (1t-FPG ≥ 126 mg/dL), and/or incomplete or implausible data in some fields were excluded. 

### 2.1. Outcomes

Maternal characteristics that were assessed included age, body mass index (BMI), gestational smoking, chronic hypertension, 1t-FPG and 2t-FPG values and insulin requirement. Perinatal outcomes included preeclampsia, defined according to the criteria of the International Society for the Study of Hypertension in Pregnancy [20], prematurity (pregnancy duration < 37 weeks), mode of delivery (vaginal or caesarean section), Apgar score at 1 and 5 min, umbilical artery pH level and admission to the neonatal intensive care unit (NICU). Birth weight was converted into a percentile using customized curves from the Spanish singleton pregnancy guidelines [21] and infants were classified as large for gestational age (LGA) or small for gestational age (SGA) if birth weight was above or below the cut-off for the 90th or 10th percentile, respectively. In addition, a composite adverse outcome was considered, including at least one of the following: preeclampsia, caesarean section, LGA, and Apgar < 7 at 1st and 5th minute or NICU admission.

### 2.2. Subgroups and Comparisons

- **Post-COVID Early-GDM** was defined using 1t-FPG ≥ 100 mg/dL, according to the recommendations of Spain [4] for the diagnosis of gestational hyperglycemia, GDM, in the first trimester. The following subgroups were identified:
  - **Post-COVID Missed Early-GDM group:** Pregnant women with Early-GDM and 1t-FPG < 100 mg/dL. This group was compared with the Non-GDM group, and
  - **Post-COVID New Early-GDM group:** Pregnant women with normal glucose tolerance and 1t-FPG ≥ 100 mg/dL. This group was compared with the Early-GDM group.
- **Post-COVID GDM was defined using 2t-FPG as diagnostic test**.
  The following subgroups were identified according to the glucose threshold recommended by Australia (1), Italy (2) Spain (4), and the United Kingdom (5) and the European Society of Endocrinology (3) (100 mg/dL) to diagnose or rule out GDM.
  - **Post-COVID Missed-GDM groups:** Pregnant women with GDM and 2t-FPG < 84 mg/dL, <92 mg/dL, <95 mg/dL, and <100mg/dL. Each of these groups was compared with the non-GDM group, and
  - **Post-COVID New GDM groups:** Pregnant women with normal glucose tolerance, Non-GDM, and 2t-FPG ≥ 92 mg/dL, ≥95 mg/dL, and >100 mg/dL. Each of these groups was compared with the GDM group.

**Figure 1:**
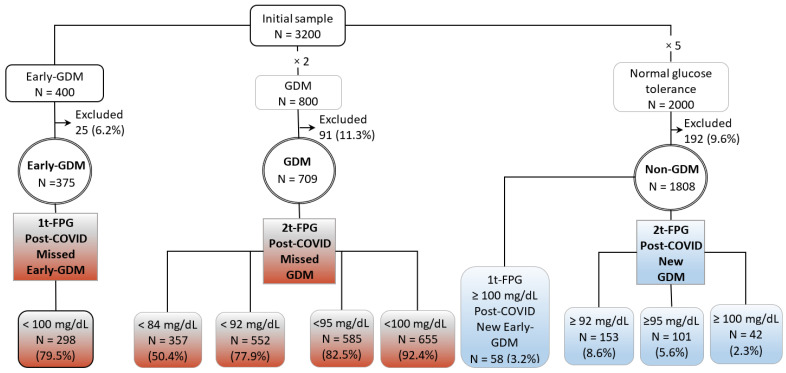
The initial and final samples included in this study. Pregnant women with gestational diabetes mellitus (GDM group), with GDM diagnosed in the first trimester, (Early-GDM group) and women with normal glucose tolerance in pregnancy (Non-GDM group), according the two-step diagnosis criteria (18), are indicated. The Post-COVID-19 Missed- and New-Early-GDM groups were identified according to the recommendations of Spain (4) for the Early-GDM diagnosis during pandemic, first trimester fasting plasma glucose (1t-FPG) < or ≥100 mg/dL, respectively. The post-COVID-Missed-GDM and post-COVID-New-GDM groups were identified according to the recommendations of Australia (1), Italy (2), Spain (3), and UK (5), FPG at 24–28 weeks of pregnancy (2t-FPG) <84, <92, <95 and <100 mg/dL, respectively or ≥92, ≥95 and ≥100 mg/dL, respectively. GDM, gestational diabetic mellitus; NDDG, National Diabetes Data Group; post-COVID, post-coronavirus disease.

Figure 1 shows these groups and the subgroups considered according to 1t-FPG and 2t-FPG diagnostic thresholds for post-COVID Early- and GDM diagnosis.

### 2.3. Statistical Methods

The normality of the date was investigated using histograms and the Kolmogorov–Smirnov test. Numerical data are shown as mean and standard deviation for parametric variables. Qualitative variables are expressed as frequencies and percentages. Differences between groups were studied using Student’s t-test. Comparison between proportions was performed using the chi-squared test and the Fisher’s exact test when any of the expected values were less than 5. 

Multivariate logistic regression was used to compare the adverse perinatal outcomes between groups, adjusting for maternal age, BMI, parity and chronic hypertension. For the selection of the adjustment variables, a backward Wald method was used (p-out = 0.1). After taking into account all of the other variables, maternal age and BMI remained in all the models of comparison between groups when the different adverse perinatal outcomes were introduced, except when prematurity and SGA were included, in which parity also remained in the models.

For statistical analysis, the pieces of software SPSS 25.0 (IBM SPSS, Armonk, New York, NY, USA) and EPIDAT 4.2 (Ministry of Health, Xunta de Galicia, Spain; Pan American Health Organization (PAHO-WHO); CES University, Medellín, Colombia) were used.

## 3. Results in Which Parity Was Also Included

From an initial sample of 3200 cases, 400 with Early-GDM, 800 with GDM and 2000 women with normal glucose tolerance, non-GDM, 25 (6.2%), 91 (11.3%), and 211 (10.5%), respectively, were excluded from each group due to incomplete or implausible data in some fields. The final sample consisted of 2873 cases, 375 with Early-GDM, 709 with GDM and 1789 pregnant women with normal glucose tolerance, non-GDM, according to the NDDG Figure 1. 

Maternal age, BMI, parity, and the rate of chronic hypertension and insulin requirement (yes or not) in the Early-GDM, GDM and Non-GDM group, as well as in the different subgroups considered, are shown in the Table 2, Table 3, Table 4 and Table 5. 

### 3.1. Diagnosis of Early-GDM during the COVID Pandemic

In the Early-GDM group, 79.5% pregnant women showed a 1t-FPG ≥ 100 mg/dL and, according to the Spain recommendation (4), they were undiagnosed (Post-COVID Missed-Early-GDM group). All maternal characteristics analysed, age, parity, BMI and the rate of chronic hypertension, were significantly higher in the post-COVID Missed-Early-GDM group than in the non-GDM group. After controlling for maternal characteristics, the rates of caesarean section, NICU admission and composite adverse perinatal outcome were higher in the Missed-Early-GDM group. 

In the non-GDM group, 13.2% women showed a 1t-FPG ≥ 100 mg/dL (Post-COVID New-Early-GDM group). No differences were found in the perinatal outcomes between the post-COVID New-Early-GDM group and the Early-GDM, after controlling for maternal characteristics Table 2.

### 3.2. Diagnosis of GDM during the COVID Pandemic

#### 3.2.1. Recommendations of Australia

According to the Australian Societies recommendations [1] to rule out GDM (2t-FPG < 84 mg/dL), 50.4% pregnant women with GDM were undiagnosed (Post-COVID-Missed-GDM group), only 22.1% were directly diagnosed (2t-FPG ≥ 92 mg/dL) and 27.5% required an OGTT (2t-FPG ≥ 84 y < 92 mg/ dL) for the diagnosis, or not, of GDM. After controlling for maternal characteristics, the rate of caesarean section was significantly higher in the Missed-GDM group than in the Non-GDM, and the rate of composite adverse perinatal outcome was 41.2% versus 35.4%, respectively, (*p* = 0.077) Table 3.

#### 3.2.2. Recommendation of Italy 

Using 2t-FPG with a threshold of 92 mg/dL as the sole criterion for the GDM diagnosis during the COVID pandemic, according to Italy (2) recommendations, the rate of pregnant women with post-COVID missed GDM was 77.9%. The rate of composite adverse perinatal outcome was higher in the Post-COVID-Missed-GDM group than in the Non-GDM. 

Considering 2t-FPG with a threshold of 92 mg/dL for Post-COVID direct diagnosis of GDM, according to Australia (1) and Italy (2) recommendations, the rate of pregnant women with new GDM was 8.6%. No differences were found in perinatal outcomes between the Post-COVID New-GDM and the GDM group. Table 3.

#### 3.2.3. Recommendation of Spain

According to the Spain recommendations for the GDM diagnosis (2t-FPG ≥ 95 mg/dL) during the pandemic, the rates of missed GDM and new GDM were 82.5% and 5.6%, respectively. 

The rate of composite adverse outcome was 41.4% in the Missed-GDM group versus 35.4% in the Non-GDM (*p* < 0.023). No differences were found in perinatal outcomes between the post-COVID New-GDM group and the GDM, after controlling for maternal characteristics, Table 4.

#### 3.2.4. Recommendations of the United Kingdom and the European Society of Endocrinologists

Finally, using 2t-FPG ≥ 100 mg/dL for post-COVID-GDM diagnosis, the rates of Missed and -New Late-GDM were 92.4% and 2.3%, respectively. 

The rate of preeclampsia, caesarean section, LGA and composite adverse perinatal outcome were significantly higher in the Post-COVID Missed-GDM group than in the Non-GDM (*p* < 0.001 and *p* < 0.02), Table 5.

## 4. Discussion 

In our study, following the recommendation of Spain for the diagnosis of early-GDM or gestational hyperglycemia in the first trimester during the COVID pandemic (1t-FPG ≥ 100 mg/dL), 79.5% of pregnant women with Early-GDM, according to an OGTT and two-step approach [18], were undiagnosed and an extra 3.2% of women with normal glucose tolerance were identified with Early-GDM. After controlling for maternal characteristics, perinatal outcomes were worse in women with missed Early-GDM than in controls. No differences were found between the post-COVID New-Early GDM group and pre-pandemic Early-GDM, although the pregnant women with Early-GDM received treatment during all pregnancy and women with New-Early-GDM did not.

Fasting plasma glucose is nowadays routinely measured during early pregnancy for overt diabetes screening, using a threshold of 126 mg/dL for the diagnosis. The ADA [22] considers the same diagnostic criteria for diabetes in the non-pregnant state than in early pregnancy and values of FPG between 100 and 126 mg/dL are accepted to diagnose hyperglycemia or prediabetes. Riskin et al. [23] showed that FPG at the first prenatal visit lower than 100 mg/dL was associated with adverse pregnancy outcomes and with an increased risk of GDM at 24–28 weeks of pregnancy; however, a clear threshold value has not been established. The IASDPG [24], according to the data observed at the second trimester in the HAPO study, recommended a 1t-FPG ≥ 92 mg/dL for the diagnosis of Early-GDM; however, this threshold has been questioned [25,26] and no other cut-off points have been proposed. In this context, the Spanish Group of Diabetes and Pregnancy [4] recommended to use FPG with a threshold of 100 mg/dL for the diagnosis of Early-GDM or gestational hyperglycemia during the COVID-19 pandemic, in addition to RPG or HbA1C, as other scientific societies proposed [1,2,3,4,5,6,7].

The determination of 2t-FPG at 24–28 weeks of pregnancy has been one of the options proposed to simplify the diagnosis of GDM during the pandemic. The thresholds considered for diagnosis range from 84 to 100 mg/dL [1,2,3,4,5,6,7]. 

According to the recommendations of Australia (1) to rule out GDM (2t-FPG < 84 mg/dL), 50% of women with GDM were post-COVID undiagnosed, 22% were directly diagnosed with GDM (2t-FPG ≥ 92 mg/dL), and 28% required an OGTT (2t-FPG ≥ 84 mg/dL and <92 mg/dL). McIntyre et al. [11] observed a lower rate of post-COVID GDM, 25%, and better perinatal outcomes in the undiagnosed pregnant women than in controls, while in our study, perinatal outcomes were worse in the post-COVID-19 missed GDM group. 

Using 2t-FPG ≥ 92 mg/dL, following the proposal of Italy [2] as a sole criterion for the diagnosis of GDM during the pandemic, the rate of women with missed GDM was 80%, and 8.6% pregnant women with normal glucose tolerance were diagnosed with new-GDM. According to the IADPSG [24], 2t-FPG ≥ 92 mg/dL (HAPO odds ratio 1.75) would diagnose more than half of GDM cases.

The rate of pregnant women with missed GDM increased to 82.6% and 92.4%, when the 2t-FPG thresholds used for the diagnosis were 95 and 100 mg/dL, according to the recommendation of Spain [4], and the United Kingdom (5) and the European Society of Endocrinologists [3], respectively. Parallel to this, the number of control women with post-COVID new-GDM decreased progressively from 5.6 to 2.3%, from the less to the most restrictive criteria. No differences were found between Post-COVID New-GDM groups and GDM, although pregnant women with pre-pandemic GDM were treated and women with new GDM were not. McIntyre et al. [11] found that the UK [5] strategies during the COVID-19 pandemic would reduce the rate of GDM diagnoses by over 80% and in consonance with our results, the woman with missed GDM had a higher frequency of adverse perinatal outcomes. 

All rates of post-COVID undiagnosed GDM are higher in our study than previously estimated by other authors [11,12,13,14]. These differences may be attributed to the criteria used for the diagnosis of GDM before the pandemic, the two-step diagnosis [18] in our study versus the less strict one-step diagnosis [24] in all previous studies [11,12,13,14]. 

We have not found any reference to the rates of pregnant women with non-GDM diagnosed with new Early-GDM and GDM according to the recommendations for the COVID-19 pandemic or their perinatal outcomes. In our study, these rates were low, 3.2% to 8.6%, but it would mean a considerable increase in the number of women diagnosed during the pandemic and unjustifiably treated. 

We are aware that our study has some limitations. The main weaknesses are the limited size of the sample, the observational retrospective design and the fact that pregnant women with Early-GDM and GDM were treated. As most of the women included were European-origin Caucasians, caution must also be applied, as the findings might not be transferable to other populations. However, our results could help to shed some light on two aspects that, to our knowledge, have not previously been considered: the rate and risks of adverse perinatal outcomes for pregnant women with Early-GDM or GDM, according to the two-step diagnostic criteria, who were not diagnosed using FPG and the recommended diagnostic thresholds proposed during the COVID-19 pandemic (50–92%), and, conversely, between 3.2% and 8.6% women with normal glucose tolerance in pregnancy were diagnosed and would be treated for GDM.

All Scientific Societies have explicitly recognised that the proposed recommendations for the diagnosis of GDM during the COVID-19 pandemic are temporary urgent measures taken during an unprecedented health situation and lacking supportive scientific evidence. 

## 5. Conclusions

In conclusion, fasting plasma glucose is not a good test for the diagnosis of GDM either in the first trimester or at 24–28 weeks. Substituting the two-step approach for the diagnosis of GDM by a fasting plasma glucose test would mean that a significant number of women at risk of adverse perinatal outcomes would not be diagnosed or treated, while others, with similar perinatal outcomes to pregnant women with normal glucose tolerance, would be diagnosed with GDM and unjustifiably treated. The pre-pandemic diagnostic criteria for GDM would be reinstated as soon as possible. 

## Figures and Tables

**Table 1:** Summary of the different recommendations for the diagnosis of gestational diabetes mellitus (GDM) during the COVID-19 pandemic.

	First Prenatal Visit. Early GDM	GDM
	Glucose Measured before 20 Weeks	Glucose Measured after 24 Weeks
**Australia** [1]	HbA1c ≥ 5.9% (41 mmol/mL)	FPG: <84 mg/dL (<4.7 mmol/L) → Non GDM FPG: 84–91 mg/dL (4.7–5 mmol/L) → OGTT FPG: ≥92 mg/dL (5.1mmol/L) → GDM
**Italy** [2]	FPG ≥ 92 mg/dL (5.1 mmol/L)	FPG ≥ 92 mg/dL (5.1 mmol/L)
**European Society of Endocrinologists** [3]	HbA1c: 5.9–6.4% (41–47 mmol/mL) or RPG: 162–200 mg/dL (9–11 mmol/L)	HbA1c ≥ 5.7% (≥ 39 mmol/L) or RPG ≥ 162 mg/dL (≥ 9 mmol/L) FPG ≥ 100 mg/dL (≥ 5.6 mmol/L)
**Spain** [4]	HbA1c: 5.9–6.4% (41–47 mmol/mL) or RPG: 165–199 mg/dL (9.2–11 mmol/L) or FPG: ≥100 mg/dL (≥5.6 mmol/L)	HbA1c ≥ 5.7% (≥39 mmol/L) or RPG: 165–199 mg/dL or FPG ≥ 95 mg/dL (≥5.3 mmol/L)
**United Kingdom** [5]	HbA1c: 5.9–6.4% (41–47 mmol/mL) or RPG: 162–200 mg/dL (9–11 mmol/L)	HbA1c ≥ 5.7% (≥39 mmol/L) or RPG ≥ 162 mg/dL (≥9 mmol/L) FPG ≥ 100 mg/dL (≥5.6 mmol/L)
**Canada** [6]		HbA1c ≥ 5.7% (≥39 mmol/L) or RPG ≥ 200 mg/dL (≥11.1 mmol/L)
**New Zealand** [7]	HbA1c ≥ 5.8% (40 mmol/mL)	FPG: <81 mg/dL (<4.5 mmol/L) → Non GDM FPG: 81–90 mg/dL (4.7–5 mmol/L) → CGT FPG: ≥90 mg/dL (≥5 mmol/L) → GDM

HbA1c: gycosylated hemoglobin A1c; FPG: fasting plasma glucose; RPG: random plasma glucose; CGT: capillary glucose testing; GDM: gestational diabetes.

**Table 2:** Maternal characteristics and perinatal outcomes in pregnant women with gestational diabetes mellitus diagnosed in the first trimester of pregnancy (Early-GDM), and with normal glucose tolerance during pregnancy (non-GDM), according to the two-step diagnosis (18) and in women with missed and new Early-GDM according to the recommendation of Spain for Early-GDM diagnosis during the COVID-19 pandemic (4) (first trimester fasting plasma glucose (1t-FPG) ≥100 mg/dL).

	Early-GDM		Non-GDM		*p*-Values	
	1t-FPG < 100 mg/dL	Total	1t-FPG ≥ 100 mg/dL	Total	Missed Early-GDM vs. Total Non-GDM	New-Early-GDM vs. Total Early GDM
	Post-COVID Missed Early-GDM		Post-COVID- New Early GDM			
	N = 298 (79.5%)	N = 375	N = 58 (3.2%)	N = 1789		
Maternal age (years)	35.2 ± 4.7	35.2 ± 4.6	31.3 ± 6.4	30.1 ± 6.0	<0.001	<0.001
BMI (kg/m^2^)	28.6 ± 6.4	29.4 ± 6.6	28.6 ± 5.6	25.7 ± 5.0	<0.001	0.341
Parity > 1, n (%)	161 (54.0)	212 (56.5)	41 (70.7)	789 (44.1)	0.002	0.046
Chronic Hypertension, n (%)	29 (9.6)	35 (9.4)	2 (3.4)	22 (1.2)	<0.001	0.184
Insulin, n (%)	144 (48.3)	189 (50.4)	0	0	-	-
**Perinatal outcomes**						
Preeclampsia, n (%)	13 (4.4)	17 (4.5)	1 (1.7)	27 (1.5)	0.152	0.360
Prematurity, n (%)	30 (10.1)	41 (10.9)	5 (8.6)	108 (6.0)	0.114	0.731
Caesarean section, n (%)	90 (30.5)	114 (30.6)	8 (13.8)	233 (13.0)	0.004	0.113
LGA, n (%)	45 (15.1)	62 (16.5)	7 (12.1)	205 (11.5)	0.648	0.725
SGA, n (%)	31 (10.4)	38 (10.1)	8 (13.8)	205 (11.5)	0.899	0.904
1m Apgar test ≤ 7, n (%)	33 (11.4)	41 (11.2)	8 (13.8)	136 (7.6)	0.068	0.553
5m Apgar test ≤ 7, n (%)	1 (0.3)	4 (1.1)	1 (1.7)	31 (1.7)	0.217	0.902
pH artery < 7, n (%)	3 (1.0)	5 (1.3)	1 (1.7)	28 (1.6)	0.329	0.860
NICU, n (%)	40 (13.4)	48 (12.8)	7 (12.1)	138 (7.7)	0.001	0.889
Composite adverse outcome n (%)	145 (48.7)	189 (50.4)	23 (39.7)	633 (35.4)	0.044	0.595

Results are shown as frequency (%) or means ± SD. BMI: body mass index; GWG: gestational weight gain; LGA: large for gestational age; SGA: small for gestational age; NICU: neonatal intensive care unit admission; vs.: versus. Composite adverse outcome: preeclampsia, caesarean, LGA, Apgar < 7 at 1st or 5th min, and/or NICU admission. *p*-values adjusted for maternal characteristics (maternal age, BMI, parity and chronic hypertension).

**Table 3:** Maternal characteristics and perinatal outcomes in pregnant women with gestational diabetes mellitus diagnosed at 24–28 weeks of pregnancy (GM) and with normal glucose tolerance during pregnancy (Non-GDM), according to the two-step diagnosis (18), and in women with post-COVID-19 missed GDM based on the Australia (1) recommendations to rule out the diagnosis of GDM (second trimester fasting plasma glucose (2t-FPG) <84 mg/dL), and perinatal outcomes in pregnant women with missed and new GDM based on the recommendations of Australia (1) and Italy (2) for the direct GDM diagnosis during the COVID-19 pandemic (2t-FPG) ≥92 mg/dL.

	Total		GDM	*p*-Values	GDM	Non-GDM	*p*-Values	
	GDM	Non-GDM	2t-FPG < 84 mg/dL	Missed- GDM vs. Total Non-GDM	2t-FPG < 92 mg/dL	2t FPG ≥ 92 mg/dL	Missed- GDM vs. Total Non-GDM	New-GDM vs. Total -GDM
			Post-COVID Missed		Post-COVID Missed	Post-COVID New-GDM		
	N = 709	N = 1789	N = 357 (50.4%)		N = 552 (77.9%)	N = 153 (8.6%)		
Maternalage (years)	34.1 ± 5.0	30.1 ± 6.0	33.7 ± 5.1	<0.001	34.1 ± 5.1	31.2 ± 5.6	<0.001	<0.001
BMI (kg/m^2^)	26.8 ± 6.0	25.7 ± 5.0	25.3 ± 5.4	0.127	26.1 ± 5.6	28.5 ± 5.3	0.227	0.001
Parity > 1, n (%)	335 (47.2)	789 (44.1)	136 (38.1)	0.040	247 (44.7)	81 (52.9)	0.806	0.212
Chronic Hypertension, n (%)	42 (6.0)	22 (1.2)	16 (4.6)	0.001	28 (5.1)	6 (3.9)	<0.001	0.421
2t-FPG (mg/dL)	84.3 ± 10.1	80.2 ± 8.2	76.3 ± 5.1	<0.001	80.2 ± 6.8	97.5 ± 6.1	0.815	<0.001
**Perinatal outcomes**								
Preeclampsia, n (%)	30 (4.2)	27 (1.5)	8 (2.2)	0.497	16 (2.9)	4 (2.6)	0.252	0.190
Prematurity, n (%)	61 (8.6)	108 (6.0)	23 (6.4)	0.938	41 (7.4)	14 (9.2)	0.325	0.925
Cesarean, n (%)	169 (23.9)	233 (13.0)	82 (23.0)	<0.001	127 (23.0)	26 (17.0)	<0.001	0.109
LGA, n (%)	107 (15.1)	205 (11.5)	46 (12.9)	0.220	78 (14.1)	27 (17.6)	0.071	0.580
SGA, n (%)	75 (10.6)	205 (11.5)	38 (10.6)	0.586	60 (10.9)	17 (11.1)	0.962	0.808
1m Apgar ≤ 7 n (%)	52 (7.5)	136 (7.6)	27 (7.8)	0.691	37 (6.9)	10 (6.5)	0.878	0.464
5m Apgar ≤ 7, n (%)	10 (1.5)	31 (1.7)	5 (1.5)	0.535	8 (1.5)	3 (1.9)	0.638	0.402
pH artery < 7, n (%)	8 (1.1)	28 (1.6)	4 (1.1)	0.800	5 (0.9)	3 (2.0)	0.551	0.351
NICU, n (%)	59 (8.3)	138 (7.7)	27 (7.6)	0.900	41 (7.4)	18 (11.8)	0.385	0.366
Composite adverse outcome n(%)	301 (42.5)	633 (35.4)	147 (41.2)	0.077	227 (41.1)	69 (45.1)	0.038	0.685

Results are shown as frequency (%) or means ± SD. BMI: body mass index; CI: confidence interval; 2t-FPG: second trimester fasting plasma glucose LGA: large for gestational age; SGA: small for gestational age; NICU: neonatal intensive care unit admission; composite adverse outcome: preeclampsia, caesarean, LGA, Apgar < 7 at 1st or 5th min, and/or NICU admission. *p*-values adjusted for maternal characteristics (maternal age, BMI, parity and chronic hypertension).

**Table 4:** Maternal characteristics and perinatal outcomes in pregnant women with gestational diabetes mellitus diagnosed at 24–28 weeks of pregnancy (GM) and with normal glucose tolerance during pregnancy (Non-GDM), according to the two-step diagnosis (18) and in pregnant women with missed and new GDM based on the recommendations of Spain (4) for GDM diagnosis during the COVID-19 pandemic, second trimester fasting plasma glucose level (2t-FPG) ≥95 mg/dL.

	GDM		Non-GDM		*p*-Values	
	2t-FPG < 95 mg/dL	Total	2t FPG ≥ 95 mg/dL	Total	Missed-GDM vs. Total Non-GDM	New-GDM vs. Total GDM
	Post-COVID Missed-GDM		Post-COVID New-GDM			
	N = 585 (82.5%)	N = 709	N = 101 (5.6%)	N = 1789		
Maternal age (years)	34.1 ± 5.0	34.1 ± 5.0	31.6 ± 5.7	30.1 ± 6.0	<0.001	<0.001
BMI (kg/m^2^)	26.2 ± 5.7	26.8 ± 6.0	28.7 ± 5.8	25.7 ± 5.0	0.078	0.003
Parity > 1, n (%)	266 (45.5)	335 (47.2)	52 (51.5)	789 (44.1)	0.566	0.457
Chronic Hypertension, n (%)	29 (5.0)	42 (6.0)	4 (4.0)	22 (1.2)	<0.001	0.636
2t-FPG (mg/dL)	80.9 ± 7.2	84.3 ± 10.1	99.8 ± 6.3	80.2 ± 8.2	0.092	<0.001
**Perinatal outcomes**						
Preeclampsia, n (%)	19 (3.2)	30 (4.2)	3 (3.0)	27 (1.5)	0.097	0.330
Prematurity, n (%)	49 (8.4)	61 (8.6)	10 (9.9)	108 (6.0)	0.067	0.870
Caesarean section, n (%)	136 (23.3)	169 (23.9)	18 (17.8)	233 (13.0)	<0.001	0.184
LGA, n (%)	83 (14.2)	107 (15.1)	19 (18.8)	205 (11.5)	0.064	0.458
SGA, n (%)	63 (10.8)	75 (10.6)	11 (10.9)	205 (11.5)	0.847	0.933
1m Apgar test ≤ 7, n (%)	38 (6.7)	52 (7.5)	8 (7.9)	136 (7.6)	0.743	0.812
5m Apgar test ≤ 7, n (%)	8 (1.4)	10 (1.5)	2 (2.0)	31 (1.7)	0.531	0.497
pH artery < 7, n (%)	5 (0.9)	8 (1.1)	2 (2.0)	28 (1.6)	0.454	0.384
NICU, n (%)	47 (8.0)	59 (8.3)	10 (9.9)	138 (7.7)	0.165	0.895
Composite adverse outcome n (%)	242 (41.4)	301 (42.5)	50 (49.5)	633 (35.4)	0.023	0.298

Results are shown as frequency (%) or means ± SD. BMI: body mass index; CI: confidence interval; 2t-FPG: second trimester fasting plasma glucose; LGA: large for gestational age; SGA: small for gestational age; NICU: neonatal intensive care unit; composite adverse outcome: preeclampsia and/or caesarean, LGA, Apgar < 7 at 1st or 5th min, NICU admission. *p*-values adjusted for maternal characteristics (maternal age, BMI, parity and chronic hypertension).

**Table 5:** Maternal characteristics and perinatal outcomes in pregnant women with gestational diabetes mellitus diagnosed at 24–28 weeks of pregnancy (GDM) and with normal glucose tolerance during pregnancy (Non-GDM), according to the two-step diagnosis (18) and in pregnant women with missed and new GDM based on the recommendations of the European Society of Endocrinologists (3) and the United Kingdom (5) for GDM diagnosis during the COVID-19 pandemic, second trimester fasting plasma glucose level (2t-FPG) ≥100 mg/dL.

	GDM		Non-GDM		*p*-Values	
	2t-FPG < 100 mg/dL	Total	2t FPG ≥ 100 mg/dL	Total	Missed-GDM vs. Total Non-GDM	New-GDM vs. Total GDM
	Post-COVID-Missed-GDM		Post-COVID-New Late GDM			
	N = 655 (92.4%)	N = 709	N = 42 (2.3%)	N = 1789		
Maternal age (years)	34.1 ± 5.0	34.1 ± 5.0	32.5 ± 4.8	30.1 ± 6.0	<0.001	0.050
BMI (kg/m^2^)	26.5 ± 5.9	26.8 ± 6.0	29.2 ± 5.6	25.7 ± 5.0	0.004	0.013
Parity > 1, n (%)	304 (46.4)	335 (47.2)	23 (54.8)	789 (44.1)	0.313	0.427
Chronic Hypertension, n (%)	38 (5.8)	42 (6.0)	1 (2.4)	22 (1.2)	<0.001	0.500
2t-FPG (mg/dL)	82.5 ± 8.3	84.3 ± 10.1	103.8 ± 8.2	80.2 ± 8.2	<0.001	<0.001
**Perinatal outcomes**						
Preeclampsia, n (%)	26 (4.0)	30 (4.2)	1 (2.4)	27 (1.5)	0.021	0.402
Prematurity, n (%)	54 (8.2)	61 (8.6)	4 (9.5)	108 (6.0)	0.067	0.957
Caesarean section, n (%)	154 (23.5)	169 (23.9)	6 (14.3)	233 (13.0)	<0.001	0.099
LGA, n (%)	99 (15.1)	107 (15.1)	10 (23.8)	205 (11.5)	0.028	0.183
SGA, n (%)	70 (10.7)	75 (10.6)	4 (9.5)	205 (11.5)	0.721	0.937
1m Apgar test ≤ 7, n (%)	47 (7.4)	52 (7.5)	3 (7.1)	136 (7.6)	0.983	0.633
5 m Apgar test ≤ 7, n (%)	10 (1.6)	10 (1.5)	-	31 (1.7)	0.611	0.999
pH artery < 7, n (%)	7 (1.1)	8 (1.1)	1 (2.4)	28 (1.6)	0.334	0.475
NICU, n (%)	55 (8.4)	59 (8.3)	3 (7.1)	138 (7.7)	0.207	0.546
Composite adverse outcome, n (%)	278 (42.4)	301 (42.5)	21 (50.0)	633 (35.4)	0.020	0.513

Results are shown as frequency (%) or means ± SD. BMI: body mass index; CI: confidence interval; 2t-FPG: second trimester fasting plasma glucose: LGA, large for gestational age; SGA, small for gestational age; NICU, neonatal intensive care unit; composite adverse outcome: preeclampsia and/or caesarean, LGA, Apgar < 7 at 1st or 5th min, NICU admission. *p*-values adjusted for maternal characteristics (maternal age, BMI, parity and chronic hypertension).

## Data Availability

The datasets generated during and/or analysed during the current study are available from the corresponding author on reasonable request.
